# Plan for 1870

**Published:** 1870-01

**Authors:** 


### PLAN FOR 1870.

It is not proposed, in making the change in the JOURNAL OF HEALTH to make any overwhelming promises of great things, of striking things, of astonishing things; there will be no effort to create a sensation. We simply state the reason for the alterations in the form, size and matter of HALL'S JOURNAL OF HEALTH. There is stealth in the idea.

It is so very difficult to get persons in health to take measures to preserve it, that not more than one person in a thousand met on the public street would be willing to subscribe for the Journal for his family; almost, if not quite, as difficult as it is to induce men in high health and worldly prosperity to give a wise and sufficient attention to the claims of religion. Hence it was thought that if there was additional reading in the Journal of a general character, short histories for children, and useful sentiments for all in reference to the conduct of life and business success, it might be taken up from time to time for the sake of these; and now and then a glance might be given to some short sentence or narration in reference to health, which might make an indelible impression on the young mind to be remembered and carried out for a life-time afterward.

Then again, it is a refreshing thought that our Journal will be always safer reading, suitable for every age and sex and sect; that there will never a line appear in it, intentionally, which will not be on the side of good government, good morals and good books; never a line in disparagement of woman, of the clergy, of the Bible, of the Sabbath-day and of Religion; on the contrary, these will be always upheld, and always revered and honored, as they ought to be, for no nation can prosper long and solidly if even one be absent.

An engraving or chromo-painting is offered to each subscriber, who pays two dollars at a time for one year's subscription. In doing this, we hope to cultivate a taste for chaste paintings and engravings, for they elevate and refine the mind, the feelings, the sentiments and the whole man, like music and the fine arts in general.

Most of the chromos offered represent various incidents in childhood and youth in such a form as to make it almost impossible for any one to look at them for a moment without emotions of lovingness which happify for the instant, and leave a man better than he was a moment before. The very names of some of these excite pleasant memories, much more the paintings themselves, such as the

| | |
|---|---|
| Broken Plate. | The Rivals. |
| Helping Grandm'a. | The Favorites. |
| Terrier and Puppies. | First Grief. |
| Hen and Chickens. | Brother's Toilet. |
| Dog and Cat. | Feeding Chickens. |
| Pillage. | Do. Parrot. |
| Defence. | Do. Squirrel. |

ENGRAVINGS.

Gen. M'Clellan on his War Horse.
Pictorial History of Old Testament.
Pictorial History of New Testament.
Sir Walter Scott in his Study at Abbottsford.
Signing the Death Warrant of Lady Jane Gray.
Capture of Major Andre.
Sir Walter Raleigh Parting with his Wife.
The Jolly Flat Boatman.
The Trapper's Last Shot.
Sparking—A beautiful engraving.
Parting—A Highland scene.
Anne Page, Slender and Shallow.

Thus will our Journal be a means of exciting pleasurable feelings in every heart, in every house where it is taken, and every day in the year; and all this for only Two Dollars, which is absolutely worse than thrown away in almost every family every year in worthless novels, in corrupting newspapers, story papers, in cigars, tobacco, spirits or in the vanities of dress, all of which perish with the using. Reader, think of this.

